# Consilience in Causation: Causal Emergence Is Found Across Measures of Causation

**DOI:** 10.3390/e27080825

**Published:** 2025-08-04

**Authors:** Renzo Comolatti, Erik Hoel

**Affiliations:** 1 Department of Psychiatry, University of Wisconsin-Madison, Madison, WI 53719, USA; comolatti@wisc.edu; 2 Allen Discovery Center, Tufts University, Medford, MA 02155, USA

**Keywords:** causality, causal emergence, information theory, counterfactuals

## Abstract

Causation is fundamentally important to science, and yet our understanding of causation is spread out across disparate fields, with different measures of causation having been proposed in philosophy, statistics, psychology, and other areas. Here we examined over a dozen popular measures of causation, all independently developed and widely used, originating in different fields. We identify a high degree of consilience, in that measures are often very similar, or indeed often rediscovered. This is because, we show, measures of causation are based on a small set of related “causal primitives”: sufficiency and necessity, or alternatively, determinism and degeneracy. These primitives are ways of capturing the degree of uncertainty inherent in causal relationships. In turn, these results help us understand the phenomenon whereby macroscales can reduce the uncertainty in causal relationships, leading to stronger causes at macroscales—an outcome known as “causal emergence”. First identified using the effective information and later the integrated information in model systems, causal emergence has been found in real data across the sciences since. But is it simply a quirk of these original measures? Using a simple model system, we demonstrate how the consilience of causation guarantees that causal emergence is commonly found in causal measures, identifying instances across all measures analyzed. This finding sets the mathematical understanding of emergence on firmer ground, opening the door for the detection of natural scales of causal interaction in complex systems, as well as assisting with scientific modeling and experimental interventions.

## 1. Introduction

Over the last few decades, a number of mathematical treatments of causation have originated from diverse scientific fields like psychology and statistics [1,2,3]. For example, in the neurosciences, measures of causation have been used to track the result of experimental interventions [4,5,6]. However, the plethora of measures and the absence of consensus on an optimal measure of causation introduces some subjectivity in terms of what counts as a cause, as individual scientists may prefer or use one measure over another.

Here we offer a novel way around this problem by showing that popular measures of causation are mathematically interrelated, behave similarly under many conditions, and are sensitive to the same fundamental core terms. Notably, all the measures we examined turned out to be based on a dual set of what we dub *causal primitives*. By showing how over a dozen measures of causation are grounded in the same primitives, we reveal there is remarkable agreement in terms of what constitutes a strong or weak cause (or more generally, a strong or weak causal relationship). This convergence, which we refer to as a form of *formal consilience* in causation, is exemplified by the fact that many of these measures collapse into actually being equivalent, despite having been proposed independently by different authors, such that the total number of unique measures ends up being smaller.

Our research on causal consilience obviates the need to arrive at a lone measure of causation that researchers must universally agree upon, but rather reveals a sphere of viable measures with significant overlap (much like the definitions of “complexity” in complex systems science [7]). Their agreement is, as we show, because measures of causation are based on a small set of “causal primitives”: *sufficiency* and *necessity*, along with their respective information-theoretic extensions, *determinism* and *degeneracy*. This indicates these terms are axiomatic to any understanding of causation.

We further demonstrate that the causal primitives are also fundamental to causal emergence, which is when a causal relationship is stronger at the macroscale than the microscale [8]. Causal emergence is grounded in the fact that macroscales can lead to noise reduction in causal relationships. Broadly, this noise is synonymous with uncertainty, which can come from different sources, and macroscales can reduce or minimize this error. In such cases, ignoring irreducible macroscale causation would “leave some causation on the table”, even though the macroscale supervenes (is fixed by) its underlying microscale [9]. Note that claims of emergence have real practical consequences. For example, causally emergent macroscales are more useful to intervene on and understand the system in question with [10]; causal emergence has been used to identify natural scales of causal interaction in opaque non-engineered systems where the scale of a system’s causal workings is unknown, like in gene regulatory networks [11]; it can also be used to find effective groupings of directed graphs and is more common in biological networks compared to technological networks [12]; it has revealed novel groupings of cellular automata rules [13]; causal emergence has been used to identify macrostates in timeseries data using artificial neural networks [14]; it can be used to identify emergent macroscales in flocking bird simulations, as well as such macroscales in human-derived fMRI data [15]. There is even some evidence that evolution selects for causal emergence, possibly because macroscales that are causally emergent have been shown to be more robust to knock-outs and attacks [16]. Such questions are relevant across the sciences; e.g., there are fundamental questions about what scale is of most importance in brain function [17,18,19] that only a scientific theory of emergence can resolve. In fact, causal emergence might even play a role in explaining how to identify the appropriate scale of the neural correlates of consciousness in the brain [20,21].

However, evidence for causal emergence has previously been confined to a small set of measures: first, the effective information [8,12,22], and then later the integrated information [20,23]. Both these measures, grounded in information theory, are designed to capture similar aspects of causation (albeit with a slightly different focus). They are related mathematically and involve similar background assumptions. Because of this, some have criticized the measures revealing causal emergence, pointing to how interventions are performed (e.g., perhaps effective information requiring a maximum-entropy intervention distribution means it is somehow invalid or assumptive [24]), as well as the meaning of effective information in general (e.g., perhaps it is somehow merely capturing “explanatory” causation rather than real causation [25]). Meanwhile, the integrated information has been criticized for being one of many possible measures [26,27], and unsubstantiated from its axioms [28]. While there are counterarguments to these specific criticisms of info-theoretic accounts of causation (e.g., recent work on grounding integrated information in axiomatic considerations [29,30]), it is a reasonable question whether causal emergence is a general phenomenon or some peculiar quirk of merely a few measures of causation and their background assumptions, as this would limit its relevancy significantly.

On the other hand, there are already some reasons to think causal emergence is indeed a broader phenomenon [31,32]. For example, recent evidence has indicated that the synergistic and unique information component of mutual information can be greater at macroscales (while the redundant information component is lower) [33], and there have been other causal emergence-based approaches to the partial information decomposition as well [32,34], which may provide alternative sufficient conditions for identifying macroscales [35].

Here we provide evidence for widespread occurrence of causal emergence across measures of causation. We show that, across over a dozen popular historical measures of causation, many drawn from different fields, causal emergence holds true under many different conditions and assumptions as to how the measures are applied. Instances of causal emergence can be detected by all of the independently derived measures of causation that we chose as worthwhile to consider. This is because the underlying causal primitives can be increased at a macroscale, i.e., macroscales can increase determinism and decreases degeneracy across measures. Therefore, all the measures of causation examined also demonstrate causal emergence (in fact, we find that effective information is the most conservative measure of those we analyzed). This is all despite the fact that macroscales are simply dimensional reductions of microscales. So while two scales may both be valid descriptions of a system, one may possess stronger causation (the interpretation of which, whether as more causal work, information, or explanation, depends on the measure of causation itself). Yet causal emergence is not trivially universal either. It is system-dependent: in many cases, specifically those without any uncertainty in microscale system dynamics, causal reduction dominates (in that causal emergence is actually negative).

First, in Section 2.2, we define causal primitives along with the formal language of cause and effect we will use throughout, which is as general as possible so as to apply to many different measures. In Section 2.3, we overview twelve independently proposed measures of causation (several of which end up being identical, as we show). In Section 3.1, we show how the behavior of the measures is based on causal primitives. In Section 3.2, we directly compare macroscales to microscales across all the causal measures, and find widespread evidence for causal emergence across all the measures.

In the Discussion, we overview how the consilience of causation we have revealed can provide a template for an objective understanding of causation and ties together the nascent but growing scientific subfield of understanding emergence mathematically.

## 2. Materials and Methods

### 2.1. Basic Probabilistic Framework

To integrate different approaches to causation within a unified formalism, we adopt a general probabilistic framework. In order to simplify our framework, and to compare and contrast measures, our formalism is applied in self-contained Markov chains, where we can always assume that the previous global state (at time *t*) caused the successor global state (at t+1). Our purpose is then in quantifying how “strong” or “powerful” or “informative” the causal relationship reflected by this state transition is.

Therefore, let Ω be a finite set representing all possible system states in such a Markov chain. We define two random variables over this space: *C*, denoting causes (typically the system state at time *t*); and *E*, denoting effects (e.g., the system state at time t+1). These are related through a transition probability P(e∣c) in a Markov process, which describes the probability of transitioning to state *e* at time t+1 given that the system was in state *c* at time *t*.

Throughout this work, we interpret P(e∣c) as an interventional probability—that is, the probability of *e* occurring when *c* is imposed via intervention. In the formalism of Pearl, this corresponds to P(e∣do(c)) [1]. The use of the do operator signifies that *c* is not merely observed but enacted, independent of its prior history. Under this assumption, the system’s dynamics are fully specified by a transition probability matrix (TPM), which encodes P(e∣do(c)) for all pairs (c,e). This perspective is shared by other causal frameworks [8,36,37], where the TPM is taken as the basic object encoding the causal model.

To compute the causal measures, one must also specify a distribution over the possible causes *C*, denoted $P_{C}\left(c\right)$, which we refer to as the *intervention distribution* [22]. This distribution represents the space of interventions used to evaluate counterfactuals—e.g., the probability of obtaining an effect *e* if a cause *c* had not occurred. In Section 2.4, we examine different choices for $P_{C}$, but for now we assume it is fixed. All quantities that follow, such as P(e∣C) and P(e∣C\c), are to be understood relative to this distribution.

Given the transition probability P(e∣c) and a choice of $P_{C}$, we can define the marginal (or “unconditioned”) probability of an effect *e*:

$$P\left(e|C\right)=\sum_{c\in \Omega }P_{C}\left(c\right)\;P\left(e|\mathrm{do}\left(c\right)\right)=\sum_{c\in \Omega }P_{C}\left(c\right)\;P\left(e|c\right),$$

where we write P(e∣C) instead of P(e) to emphasize its dependence on the intervention distribution. For readability, we omit the “do” notation hereafter, with the understanding that all conditionals P(e∣C) are interventional.

To evaluate counterfactuals—such as the probability of *e* given that *c* did not occur—we define a renormalized distribution over the remaining causes C\c:

$$P_{C\backslash c}\left(c^{\prime }\right)=\frac{P_{C}\left(c^{\prime }\right)}{1-P_{C}\left(c\right)}\;\mathrm{for}\;c^{\prime }\in \Omega \backslash c,$$

This allows us to compute the counterfactual probability:

$$P\left(e|C\backslash c\right)=\sum_{c^{\prime }\in \Omega \backslash c}P_{C\backslash c}\left(c^{\prime }\right)\;P\left(e|c^{\prime }\right).$$

This formal setup provides the framework for defining the causal primitives—sufficiency, necessity and others—that we introduce in the following section.

### 2.2. Causal Primitives

#### 2.2.1. Sufficiency and Necessity

Here we propose causation should be viewed not as an irreducible single relation between a cause and an effect but rather as having two dimensions: sufficiency and necessity [1,38].

**Figure 1:**
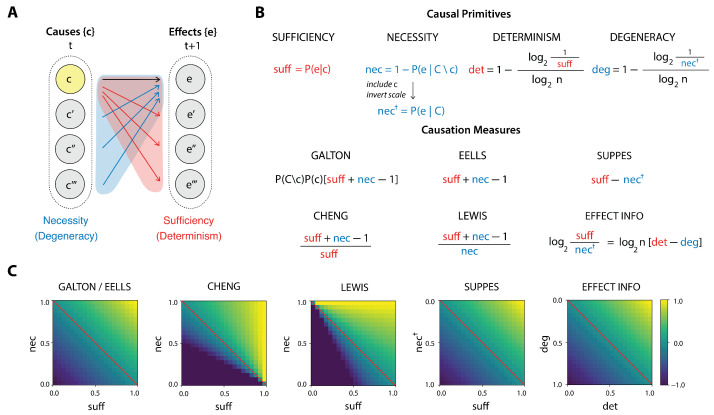
**Causal primitives and causation measures.** (**A**) Schematic representation of causation as a relation between occurrences (or events) connecting a set of causes to a set of effects. Each individual candidate cause ($c,c^{\prime },\ldots$) and candidate effect ($e,e^{\prime },\ldots )$ is depicted in a circle, while their sets *C* and *E* are marked as the enclosing dotted line. Causes and effects are assumed to be temporally ordered, with the former preceding the latter, hence are indexed at a time *t* and a time t+1, respectively. Given a pair of a candidate cause *c* and a candidate effect *e*, the relation between *c* and *e* can be analyzed in terms of the causal primitives of sufficiency and necessity. On one hand, one can assess whether *c* is *sufficient* to bring about *e*, or whether *c* can instead transition to other effects in *E* (region shaded red); on the other hand, one can ask whether *c* is *necessary* for *e* to be obtained, or instead whether other causes in *C* could also produce *e* (region shaded blue). (**B**) The functional dependence of the causation measures on the causal primitives is highlighted (sufficiency and determinism in red, necessity and degeneracy in blue). On the top are the formulas of the causal primitives, and on the bottom the formulas of the causation measures written in terms of the causal primitives (except the bit-flip measure). (**C**) Behavior of the causation measures as a function of the causal primitives (using n=2). Effective information is shown as a function of det/deg in order to highlight the resemblances with the other measures.

For any cause *c*, we can always ask, on one hand, how sufficient *c* is for the production of an effect *e*. A sufficient relation means that whenever *c* occurs, *e* also follows (Figure 1A, red region). Separately, we can also ask how necessary *c* is to bring about *e*, that is, whether there are different ways than through *c* to produce *e* (Figure 1A, blue region). Yet these properties are orthogonal: a cause *c* may be sufficient to produce *e*, and yet there may be other ways to produce *e*. Similarly, *c* may only sometimes produce *e*, but is the only way to produce it. Thus, causation occurs when sufficiency and necessity between a cause *c* and an effect *e* are jointly obtained. If this conception is correct, causal measures should then aim to quantify the degree of the joint presence of this two aspects.

As we will show, popular measures of causation indeed almost always put these two causal primitives in some sort of relationship (e.g., a difference or a ratio). This ensures such measures are mathematically quite similar, indeed, sometimes unknowingly identical. This consilience across measures leads us to refer to sufficiency and necessity as *causal primitives* of causation.

Let us now define the primitives formally. To begin, we define the sufficiency of the cause *c* as the probability:

suff(e,c)=P(e∣c)

This increases as *c* is more capable of bringing about *e*, reaching 1 when *c* is fully sufficient to produce *e*. While in classical logic, sufficiency (and necessity) are binary, absolute relations, here we have graded degrees of sufficiency (e.g., a cause might bring about its effect only some of the time), reflecting the probabilistic treatment of causation.

Comparably, the necessity of the cause for the effect we define as the probability:

nec(e,c)=1−P(e∣C\c)

This gives the inverse probability of *e* occuring given that something other than *c* occurred. Necessity is 1 when *c* is absolutely necessary for *e*. In such cases there is no other candidate cause but *c* that can produce *e*. Note that unlike sufficiency, some definition of counterfactuals needs to be made explicit for the calculation of necessity (more on this in later sections, where possible counterfactuals are represented as viable interventions).

#### 2.2.2. Determinism and Degeneracy

The two causal primitives of sufficiency and necessity each have an extension from the probabilistic to the information-theoretic setting; namely, the determinism and degeneracy coefficients [8]. These extensions allow for quantifying causation in terms of how uncertainty and noise constrains the state space of causes and effects.

We can define determinism as the opposite of noise (or randomness); that is, the certainty of causal relationships. Specifically, it is based on the entropy of the probability distribution of the effects given the occurrence of a cause:

$$H\left(E|c\right)=\sum_{e\in \Omega }P\left(e|c\right)\log_{2}\frac{1}{P(e|c)}$$

This entropy term is zero if a cause has a single deterministic effect (with P(e∣c)=1), and the entropy is maximal, i.e., $\log_{2}n$, if a cause has a totally random effect (i.e., its effects are uniformly distributed as $\frac{1}{n}$). We therefore define the determinism of a cause *c* to be $\log_{2}\left(n\right)-H\left(e|c\right)$. Note that determinism is based on sufficiency, with P(e∣c) as the central term.

To see the difference between sufficiency and determinism, consider a system of four states Ω={a,b,c,d}, wherein state *a* transitions to the other states *b*, *c*, or *d*, and also back to itself, *a*, with probability $\frac{1}{4}$ each. The sufficiency of each individual transition from *a* (e.g., to *b*) is $\frac{1}{4}$, indicating the probability that *a* produces that specific effect. However, the determinism of *a* is zero, because the entire distribution P(e∣a) is uniform, yielding maximal entropy. In other words, knowing that the system was in state a tells us nothing about what state will follow—it is indistinguishable from random selection.

This illustrates that sufficiency captures the strength of a specific transition, while determinism reflects how concentrated or selective the entire effect distribution is for a given cause (although the contribution of each transition to the determinism term can be calculated). And unlike sufficiency, the determinism term is influenced by the number of considered possibilities (i.e., size of the state space). Generally, we normalize the term to create a determinism coefficient that ranges, like sufficiency, between 0 (fully random) and 1 (fully deterministic), for a given cause:

$$det\left(c\right)=1-\frac{H(E|c)}{\log_{2}n}$$

And with this in hand, we can define a determinism coefficient for individual transitions as

$$det\left(e,c\right)=1-\frac{\log_{2}\frac{1}{P(e|c)}}{\log_{2}n}$$

as well as a system-level determinism coefficient by averaging across all possible causes:

$$det=\sum_{c\in \Omega }P\left(c\right)\;det\left(c\right)=\sum_{e,c\in \Omega }P\left(e,c\right)\;det\left(e,c\right)=1-\frac{\sum_{c\in \Omega }P\left(c\right)\;H\left(e|c\right)}{\log_{2}n}$$

In turn, degeneracy is the information-theoretic extension of necessity. Essentially, while determinism captures how targeted effects are to their causes, degeneracy captures how targeted causes are to their effects (see Figure 1A). It is also based on an entropy term:

$$H\left(E|C\right)=\sum_{e\in \Omega }P\left(e|C\right)\log_{2}\frac{1}{P(e|C)}$$

Here, instead of P(e|c) (sufficiency), the central term is P(e|C). To see the connection of this term to necessity (1−P(e∣C\c)), we first note that degeneracy is inversely related to necessity. Second, while necessity asks whether e depends uniquely on a particular c, degeneracy reflects whether many causes tend to converge on the same effect. The quantity P(e∣C) thus represents a “softened” or average form of P(e∣C\c), accounting for all causes. We are considering whether *e* can be produced not just in the absence of *c*, but all the ways, including via *c* itself, that *e* can occur (we write $nec^{†}\left(e\right)=P\left(e|C\right)$ to emphasize its relationship to nec(e,c)=P(e∣C\c)).

Proceeding analogously as before, we can define the degeneracy coefficient of an individual effect e as

$$deg\left(e\right)=1-\frac{\log_{2}\frac{1}{P(e|C)}}{\log_{2}n}$$

This quantity is maximal (equal to 1) when P(e ∣ C) = 1, and minimal (equal to 0) when $P\left(e|C\right)=\frac{1}{n}$, i.e., when the effect is uniformly probable across causes. Using this, the system-level degeneracy can be written as the expectation over effects:

$$deg=\sum_{e\in \Omega }P\left(e|c\right)\;deg\left(e\right)=1-\frac{H(e|C)}{\log_{2}n}$$

Degeneracy is zero when no effect has a greater probability than any other (assuming an equal probability across the full set of causes). Degeneracy is high if certain effects are “favored”, in that more causes lead to them (and therefore those causes are less necessary).

### 2.3. Measures of Causation

In the following section, we show how the basic causal primitives of sufficiency and necessity (or their info-theoretic alternatives, i.e., determinism and necessity) underlie the independent popular measures of causation we have examined. For the probabilistic measures we build on the compendium made by Fitelson and Hitchcock [39].

#### 2.3.1. Humean Constant Conjunction

One of the earliest and most influential approaches to a modern view of causation was David Hume’s regularity account. Hume famously defined a cause as “an object, followed by another, and where all the objects, similar to the first, are followed by objects similar to the second” [40]. In other words, causation stems from patterns of succession between events [41].

Overall, the “constant conjunction” of an event *c* followed by an event *e*, would lead us to *expect e* once observing *c*, and therefore to infer *c* to be the cause of *e*. There are a number of modern formalisms of this idea. Here we follow Judea Pearl, who interprets Hume’s notion of “regularity of succession” as amounting to what we today call correlation between events [1]. This can be formalized as the observed statistical covariance between a candidate cause *c* and effect *e*:

Cov(X,Y)=E(XY)−E(X)E(Y)

If we substitute the indicator function $X_{c}$ (and $Y_{e}$), which is 1 if *c* (respectively, *e*) occurs and 0 otherwise, in the equation above we obtain

$$\begin{array}{cc} Cov(X_{c},Y_{e}) & =P(c,e)-P(c)P(e)\\ & =P\left(c\right)P\left(e|c\right)-P\left(c\right)[P\left(c\right)P\left(e|c\right)+P\left(\bar{c}\right)P\left(e|C\backslash c\right)]\\ & =P(e|c)P(c)[1-P(c)]+P(c)P(C\backslash c)P(e|C\backslash c)\\ & =P(e|c)P(c)P(C\backslash c)+P(c)P(C\backslash c)P(e|C\backslash c)\\ & =P(c)P(C\backslash c)[P(e|c)-P(e|C\backslash c)]) \end{array}$$

where we have used the fact that P(e∣C)) can be decomposed into two weighted sums, i.e., over *c* and over C\c. Following others’ nomenclature [2], we refer to the observed statistical covariance that captures Humean conjunction as the “Galton measure” of causal strength, since it resembles the formalism for heredity of traits in biology. This expression can be rewritten to show its dependence on the causal primitives:

$${CS}_{Galton}\left(e,c\right)=P(c)P(C\backslash c)[P(e|c)-P(e|C\backslash c)]P(c)P(C\backslash c)[suff(e,c)+nec(e,c)-1]$$

While this does not imply a strict reduction to the primitives—since it includes a specific weighting by P(C)P(C\c)—it illustrates that both sufficiency and necessity jointly influence the Galton measure. It is worth noting that, even though it is considered one of the simplest (and incomplete) notions of causation, the regularity account of causation can be stated in terms of the underlying causal primitives.

#### 2.3.2. Eells’s Measure of Causation as Probability Raising

Ellery Eells proposed that a condition for *c* to be a cause of *e* is that the probability of *e* in the presence of *c* must be higher than its probability in its absence: P(e∣c)>P(e∣C\c) [42]. This can be formalized in a measure of causal strength as the difference between the two quantities:

$$CS_{Eells}=P\left(e|c\right)-P\left(e|C\backslash c\right)=suff\left(e,c\right)+nec\left(e,c\right)-1$$

When $CS_{Eells}<0$, the cause is traditionally said to be a negative or preventive cause [41], or in another interpretation, such negative values should not be considered a cause at all [3].

#### 2.3.3. Suppes’s Measure of Causation as Probability Raising

Another notion of causation as probability raising was defined by Patrick Suppes, a philosopher and scientist [43]. Translated into our formalism, his measure is

$${CS}_{Suppes}\left(c,e\right)=P\left(e|c\right)-P\left(e|C\right)=suff\left(e,c\right)-nec^{†}\left(e\right)$$

The difference between the ${CS}_{Eells}$ and ${CS}_{Suppes}$ measures involves a shift from measuring how causally *necessary c* is for *e*—whether it can be produced by other causes than *c*—to assessing how *degenerate* is the space of ways to bring *e* about. Both are valid measures, and in fact turn out to be equivalent in some contexts [39].

#### 2.3.4. Cheng’s Causal Attribution

Patricia Cheng has proposed a popular psychological model of causal attribution, where reasoners go beyond assessing pure covariation between events to estimate the “causal power” of a candidate cause producing (or preventing) an effect [44]. In her account, the causal power of *c* to produce *e* is given by

$$CS_{Cheng}\left(c,e\right)=\frac{P(e|c)-P(e|C\backslash c)}{1-P(e|C\backslash c)}=\frac{suff(e,c)+nec(e,c)-1}{nec(e,c)}$$

Cheng writes: “The goal of these explanations of P(e∣c) and P(e∣C\c) is to yield an estimate of the (generative or preventive) power of *c*”. While originally proposed as a way to estimate causes from data based off of observables, it is worth noting that, in our application of this measure, we have access to the real probabilities given by the transition probability matrix P(e∣c), and the measure therefore yields a true assessment of causal strength, not an estimation.

#### 2.3.5. Good’s Measure of Causation

I. J. Good gave not only the earliest explicit measure of causal power (according to [45]), but sought to derive a unique quantitative measure starting from general assumptions: “The main result is to show that, starting from very reasonable desiderata, there is a unique meaning, up to a continuous increasing transformation, that can be attached to ‘the tendency of one event to cause another one’” [46]. Good’s measure corresponds to the Bayesian ‘weight of evidence’ against *c* if *e* does not occur:

$$CS_{Good}\left(c,e\right)=\log_{2}\frac{1-P(e|C\backslash c)}{1-P(e|c)}=\log_{2}\frac{nec(e,c)}{1-suff(e,c)}$$

#### 2.3.6. Lewis’s Counterfactual Theory of Causation

Another substantive and influential account of causation based on counterfactuals was given by philosopher David Lewis [47]. In its basic form, Lewis’s account states that if events *c* and *e* both occur, then *c* is a cause of *e* if, had *c* not occurred, *e* would not have occurred. Lewis also extended his theory for “chancy worlds”, where *e* can follow from *c* probabilistically [48].

Following [2], who interpret Lewis’s own remarks, we formalize his conception of causal strength as the ratio

$$\frac{P(e|c)}{P(e|C\backslash c)}$$

Lewis’s ratio-based formulation expresses how much more likely the effect *e* is in the presence of *c* than in its absence. This definition is also known as “relative risk”: “it is the risk of experiencing *e* in the presence of *c*, relative to the risk of *e* in the absence of *c*” [2]. This measure can be normalized to obtain a measure ranging from −1 to 1 using the mapping p/q→(p−q)/p as

$$CS_{Lewis}\left(c,e\right)=\frac{P(e|c)-P(e|C\backslash c)}{P(e|c)}=\frac{suff(e,c)+nec(e,c)-1}{suff(e,c)}$$

Again we see that Lewis’s basic notion, once properly formalized, is based on the comparison of a small set of causal primitives. Also note that these definitions do not rely on a specification of a particular possible world. In other work, Lewis specifies that the counterfactual not-*c* is taken to be the closest possible world where *c* did not occur. That notion, which specifies a rationale for how to calculate the counterfactual, is formalized in Section 2.3.8.

#### 2.3.7. Judea Pearl’s Measures of Causation

If our claim for consilience in the study of causation is true, then authors should regularly rediscover previous measures. Indeed, this is precisely what occurs. Consider Judea Pearl, who in his work on causation has defined the previous measures ${CS}_{Eells}$, ${CS}_{Lewis}$, and ${CS}_{Cheng}$ (in some of these terms apparently knowingly, in others not).

Within his structural model semantics framework [1], he defines the “probability of necessity” as the counterfactual probability that *e* would not have occurred in the absence of *c*, given that *c* and *e* did in fact occur, which in his notation is written as $\mathrm{PN}=P({\bar{e}}_{\bar{c}}|c,e)$ (where the bar stands for the complement operator, i.e., $\bar{c}=C\backslash c$). Meanwhile, he defines the “probability of sufficiency” as the capacity of *c* to produce *e* and it is defined as the probability that *e* would have occurred in the presence of *c*, given that *c* and *e* did not occur: $\mathrm{PS}=P(e_{c}|\bar{c},\bar{e})$.

Finally, both aspects are combined to measure both the sufficiency and the necessity of *c* to produce *e* as $\mathrm{PNS}=P(e_{c},{\bar{e}}_{\bar{c}})$, such that the following relation holds: $\mathrm{PNS}=P\left(e,c\right)\mathrm{PN}+P\left(\bar{e},\bar{c}\right)\mathrm{PS}$.

In general, these quantities require a structural model to be evaluated. However, in special cases—specifically under assumptions such as exogeneity and monotonicity—Pearl shows that simplified expressions for them can be derived:

PNS=P(e∣c)−P(e∣C\c)

$$\mathrm{PN}=\frac{P(e|c)-P(e|C\backslash c)}{P(e|c)}$$

$$\mathrm{PS}=\frac{P(e|c)-P(e|C\backslash c)}{1-P(e|C\backslash c)}$$

In this setting, these measures reduce, respectively, to ${CS}_{Eells}$, ${CS}_{Lewis}$, and ${CS}_{Cheng}$, as noted by [2]. That is, within his broad framework, Pearl independently rediscovered previous measures.

Finally, to avoid terminological overlap, we reserve the terms “sufficiency” and “necessity” for their simpler probabilistic definitions (i.e., P(e∣c) and 1−P(e∣C\c)), and refer to Pearl’s measures by name or by their original references to preserve their distinction and provenance.

Overall, this consilience should increase our confidence that measures based on the combinations of causal primitives are good candidates for assessing causation.

#### 2.3.8. Closest-Possible-World Causation

As stated previously, David Lewis traditionally gives a counterfactual theory of causation, wherein the counterfactual is specified as the closest possible world where *c* did not occur [47]. In order to formalize this idea, we need to add further structure beyond solely probability transitions. That is, such a measurement requires a notion of distance between possible states of affairs (or “worlds”). One simple way to achieve this is to use binary labels of states to induce a metric using the Hamming distance [49], which is the number of bit flips needed to change one binary string into the other. In this way we induce a metric in a state space so that we can define Lewis’ notion of a closest possible world:

$$D_{H}\left(x,y\right)=\sum_{i}^{N}\left|x_{i}-y_{i}\right|$$

where *x* and *y* are two state labels with *N* binary digits (e.g., x=0001 and y=0010, N=4, such that $D_{H}\left(x,y\right)=2$). With such a distance notion specified, the counterfactual taken as the “closest possible world” where *c* did not occur is given by

$${\bar{c}}_{\mathrm{CPW}}=\underset{c’\in \Omega ,c’\neq c}{\arg\min};D_{H}\left(c,c’\right)$$

And with this in hand, we can define another measure based closely on Lewis’s account of causation as reasoned about from a counterfactual of the closest possible world:

$$CS_{Lewis\;CPW}=\frac{P\left(e|c\right)-P\left(e|{\bar{c}}_{CPW}\right)}{P(e|c)}$$

#### 2.3.9. Bit-Flip Measures

Another measure that relies on a notion of distance between states is the idea of measuring the amount of difference created by a minimal change in the system. For instance, the outcome of flipping of a bit from some local perturbation. In [50] such a measure is given as “the average Hamming distance between the perturbed and unperturbed state at time t+1 when a random bit is flipped at time *t*”. While originally introduced with an assumption of determinism, here we extend their measure to non-deterministic systems as

$$CS_{bit-flip}\left(e,c\right)=\frac{1}{N}\sum_{i}^{N}\sum_{e^{\prime }\in \Omega }P\left(e^{\prime }|c_{\left[i\right]}\right)D_{H}\left(e,e^{\prime }\right)$$

where $c_{\left[i\right]}$ corresponds to the state where the $i^{th}$ bit is flipped (e.g., if c=000, then $c_{\left[3\right]}=001$).

#### 2.3.10. Actual Causation and the Effect Information

Recently a framework was put forward [3] for assessing actual causation on dynamical causal networks, using information theory. According to this framework, a candidate cause must raise the probability of its effect compared to its probability when the cause is not specified (again, we see similarities to previous measures). The central quantity is the *effect information*, given by

$$ei\left(c,e\right)=\log_{2}\frac{P(e|c)}{P(e|C)}=\log_{2}n\left[det\left(e,c\right)-deg\left(c\right)\right]$$

Note that the effect information is actually just the log of ${CS}_{Lewis_{II}}$, again indicating consilience as measures of causation are rediscovered by later authors. It is also the individual transition contribution of the previously defined “effectiveness” given in previous work on causal emergence [8].

The effect information is thus, on one hand, a bit-measure version of the probabilistic Suppes measure, and on the other, a non-normalized difference between degeneracy and determinism.

#### 2.3.11. Effective Information

Effective information (EI) was first introduced by Giulio Tononi and Olaf Sporns as a measure of causal interaction, in which random perturbations of the system are used in order to go beyond statistical dependence [51]. It was rediscovered without reference to prior usage and called “causal specificity” [52].

The effective information is simply the expected value of the effect information over all the possible cause–effect relationships of the system:

$$EI=\sum_{e\in \Omega ,c\in \Omega }P\left(e,c\right)ei\left(c,e\right)=\log_{2}n\left[det-deg\right]$$

As a measure of causation, the EI captures how effectively (deterministically and uniquely) causes produce effects in the system, and how selectively causes can be identified from effects [8].

Effective information is an assessment of the causal power of *c* to produce *e*—as measured by the *effect information*—for all transitions between possible causes and possible effects, considering a maximum-entropy intervention distribution on causes (the notion of an intervention distribution is discussed in the next section). More simply, it is the non-normalized difference between the system’s determinism and degeneracy. Indeed, we can normalize the effective information by its maximum value, $\log_{2}n$, to obtain the *effectiveness* of the system:

$$eff=det-deg=\frac{EI}{\log_{2}n}$$

### 2.4. Intervention Distributions

As we have seen, measures of causation, which can be interpreted as “strength” or “influence” or “informativeness” or “power” or “work” (depending on the measure) are based on a combination of causal primitives. However, both the calculations of the measures themselves, as well as the causal primitives, involve further background assumptions in order to apply them.

Luckily, there are tools to formalize the issue. Previous research has introduced a formalism capable of dealing with this issue in the form of an *intervention distribution* [22]. An intervention distribution is a probability distribution over possible interventions (which may be entirely hypothetical) that a modeler or experimenter considers. Effectively, rather than considering a single do(x) operator [1], it is a probability distribution over some applied set of them. The intervention distribution fixes $P_{C}\left(c\right)$, the probability of causes, which is in fact necessary to calculate all the proposed causal measures. This can also be conceptualized as the space of available counterfactuals, where counterfactuals are equivalent to hypothetical interventions.

To give an intuition pump for how we apply intervention distributions and how those also represent counterfactuals in our framework: consider a simple causal model that details how a light switch controls a light bulb. The model consists of two binary variables, each with two states, {UP, DOWN} and {ON, OFF}, respectively. Suppose the system is currently in the state where the switch is UP and the light is ON. To assess the necessity of switch = UP for light = ON, we apply an intervention that sets the switch to DOWN and observe the outcome. If the light bulb continues to be ON when the switch is changed to DOWN, then switch = UP is not necessary for light = ON. Conversely, if the bulb turns OFF, this supports the necessity of switch = UP for the effect. In this case, intervening to set switch = DOWN yields P(ON∣do(DOWN))=0, so the necessity is 1−P(ON∣do(DOWN))=1. This relationship can be encoded in a transition matrix, wherein P(ON∣UP)=1 and P(OFF∣DOWN)=1. More generally, calculating necessity corresponds to evaluating 1−P(e∣C\c), where the effect is tested under counterfactual interventions excluding the cause in question.

However, once we move beyond simple binary cases and instead face a system where many distinct counterfactual states are possible, a further question arises: How should these alternative states be weighed relative to the actual one? Are all possible alternative causes equally relevant? Should some be prioritized over others? This is precisely the role of the intervention distribution: it specifies how the counterfactual space is explored and allows us to define quantities like P(e∣C) or P(e∣C\c) in a principled way.

We point out that a modeler or experimenter essentially has three choices for specifying an intervention distribution. The first, and most natural, is the *observational distribution*. Sometimes also called the “observed distribution”, in the dynamical systems we consider this corresponds to the stationary distribution over system states, obtained as the long-run limit of applying the transition matrix *T* (encoded in P(e∣c)) to an initial distribution $\mu_{0}$:

$$P_{\mathrm{obs}}\left(c\right)=\lim_{n\to \infty }\mu_{0}\left(c\right)T^{n}$$

Intuitively, this is the distribution the system converges to under its own dynamics. Equivalently, it satisfies the fixed-point equation π=πT, with $\sum_{c}\pi \left(c\right)=1$. In this case, P(C) is entirely determined by the system’s endogenous dynamics.

However, this choice suffers from serious problems—indeed, much has been made of the fact that analyzing causation must explicitly be about what *did not* happen, i.e., departures from dynamics, and the observational distribution misses this [53]. As an example, a dynamical system with point attractors has no causation under this assumption, nor does a cycle of COPY gates which all are in the same state. This is because the gain from mere observation to perturbing or intervening is lost when the intervention distribution equals the observational distribution. Finally, it is worth noting that definable stationary distributions rarely exist in the real world.

To remedy this, measures of causation often implicitly assume the second choice: an unbiased distribution of causes over Ω, totally separate from the dynamics of the system. In its simplest form, this is described as a *maximum-entropy interventional distribution*:

$$P_{maxent}\left(c\right)=\frac{1}{n}$$

where |Ω|=n. The maximum-entropy distribution has been made explicit in the calculations of, for instance, integrated information theory [36] or the previously described effective information of Section 2.3.11 [51]. There are a number of advantages to this choice, at least when compared to the observational distribution. First, it allows for the appropriate analysis of counterfactuals. Second, it is equivalent to randomization or noise injection, which severs common causes. Third, it is the maximally informative set of interventions (in that maximum entropy has been “injected” into the system).

However, it also has some disadvantages. Using a maximum-entropy intervention distribution faces the difficulty that if Ω is too large, it might be too computationally expensive to compute. More fundamentally, using $P_{maxent}\left(c\right)$ can lead to absurdity. To give a classic example: you go away and ask a friend to water your plant. They do not, and the plant dies. Counterfactually, if your friend had intervened to water the plant, it would still be alive, and therefore your friend not watering the plant caused its death. However, if the Queen of England had intervened to water the plant, it would also still be alive, and therefore it appears your plant’s death was caused just as much by the Queen of England. That is, $P_{maxent}\left(c\right)$, taken literally, involves very distant and unlikely possible states of affairs. However, in cases where the causal model has already been implicitly winnowed to be over events that are considered likely, related, or sensible—such an already defined or constructed or bounded causal model, like a set of connected logic gates, gene regulations, or neuronal connections—$P_{maxent}\left(c\right)$ allows for a clear application and comparison of measures of causation.

We point out there is a third possible construction of an intervention distribution. This is to take a local sampling of the possible world space (wherein locality is distance in possible worlds, states of affairs, the state space of the system, or even based on some outside non-causal information about the system). There are a number of measures of causation that are based on the idea of a *local intervention distribution*. E.g., one of the earliest and most influential is David Lewis’s idea of using the closest possible world as the counterfactual by which to reason about causation (Section 2.3.8). Other examples that implicitly take a local intervention approach include the bit-flip measure [50] of Section 2.3.9, as well as the “causal geometry” extension of effective information in continuous systems [10]. We formalize the assumptions behind these approaches as representing choosing a local intervention distribution to evaluate counterfactuals, which are then possible states of affairs that are similar (or “close”) to the current state or dynamics of the system, but still range across a different set from the observed distribution.

For example, to calculate Lewis’s measure, we can compute locality using the Hamming distance [49]. Rather than simply picking a single possible counterfactual $\bar{c}\in \Omega$ (which in Lewis’s measure would be only the closest possible world from Section 2.3.8), we can instead create a local intervention distribution which is a local sampling of states of affairs where *c* did not occur (i.e., the local set of possible worlds). This is equivalent to considering all states which are a Hamming distance less or equal to Δ from the actual state:

$$P_{local(c^{*})}\left(c\right)=\left\{\begin{array}{cc} \frac{1}{n_{\Delta }}, & \mathrm{if}\;c\in \Theta_{c^{*}}\\ 0 & \mathrm{otherwise} \end{array}\right.$$

$$\Theta_{c^{*}}=\left\{s\in \Omega |D_{H}\left(s,c\right)\leq \Delta \right\}$$

where $n_{\Delta }=\left|\Theta_{c^{*}}\right|$. For example, if we want to locally intervene within a distance Δ=1 around an actual state $c^{*}=001$, then $\Theta_{c^{*}}=\left\{001,101,011,000\right\}$ and $n_{\Delta }=4$, so that the intervention distribution is 1/4 over the four states and 0 elsewhere.

We note that local interventions avoid many of the challenging edge cases of measuring causation (albeit they do not automatically solve the question of “How local is correct?”). Therefore, we use local interventions for our main text and figures to highlight their advantages. But in our full analysis we take an exhaustive approach and consider all three choices of intervention distributions for the dozen measures. Our results reveal that (a) across the three choices of applicability of the measures regarding “What counts as a counterfactual or a viable intervention?”, the measures still behave quite similarly, and also (b), in fact, instances of causal emergence, as we will show, occur across different choices of intervention distributions. In other words, while there is always some subjectivity around assessing causation based on background assumptions or even the chosen measure, subjectivity is not the source of causal emergence.

### 2.5. Model System

**Figure 2:**
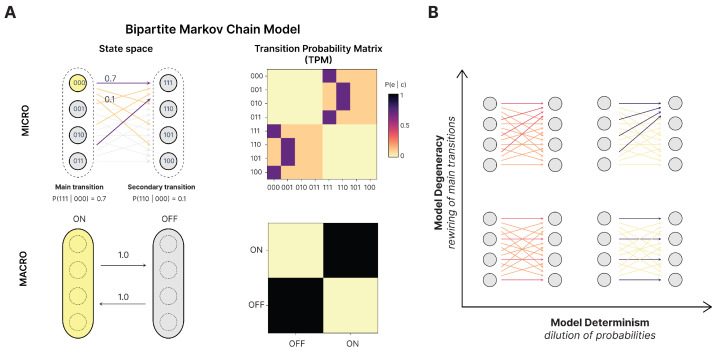
**Bipartite Markov chain model for studying causal measures.** (**A**) In this model, the system stochastically transitions between two macro-groups of microstates. The example depicts a state space with 8 microstates (**top**), partitioned into macrostates ON={000,001,010,011} and OFF={111,110,101,100} (**bottom**). A main transition from state 000 to 111 and a secondary transition from 000 to 110 are highlighted; both instantiate the same ON-to-OFF macro-transition. The transition probability matrix (TPM) at the microscale encodes the full state–state mapping (top right), while coarse-graining yields a fully deterministic macro TPM (bottom right). (**B**) State space for the bipartite model under varying levels of *determinism* (horizontal axis, manipulated via probability dilution) and *degeneracy* (vertical axis, manipulated via main-transition rewiring).

In order to examine the behavior of measures of causation presented in the previous section, we make use of a simple model. It was chosen because it allows us to parametrically vary the causal primitives of determinism (det) and degeneracy (deg) in order to see how the measures of causation change under uncertainty, and achieves this while keeping the macroscale probabilities constant under these changes. We make use of a simple bipartite Markov chain model where the microstates of the system oscillate back and forth between two groups (Figure 2). As we show in the next section, the bipartite structure ensures that when the microstate transitions are coarse-grained into ON and OFF macro-groups, the resulting macro-transition probability matrix (TPM) is a fully deterministic system: the system transitions from ON to OFF and vice versa with probability 1. Moreover, we can vary these bipartite connections to increase either the determinism (by concentrating the probability mass towards a single state in each group, approaching p=1) or the degeneracy (by increasing the overlap of state transitions, such that transitions converge in their targets). This allows us to apply the measures of causation under different amounts of uncertainty and different types of uncertainty (like indeterminism vs. degeneracy) and later to also examine causal emergence in such regimes as well. Figure 2A shows the system’s state space and transition probability matrix; panel B illustrates representative configurations across the determinism–degeneracy parameter space. A detailed description of the bipartite model, as well as how we vary these parameters, can be found in Appendix A.1.

### 2.6. Causal Emergence

To identify cases of causal emergence, traditionally a microscale and further set of candidate macroscales must be defined (it should be noted that the theory is scale-relative, in that one can start with a microscale that is not necessarily some fundamental physical microscale). In neuroscience, for instance, the “microscale” may be the scale of individual synapses. A macroscale is some dimensional reduction of the microscale, like coarse-graining (an averaging) [8] or black-boxing (only including a subset of variables in the macroscale) [54], or more generally just any summary statistic that recasts the system with less parameters while preserving the dynamics as much as possible [12,32]. e.g., in the neurosciences a macroscale may be a local field potential or neuronal population or even entire brain regions. Previous research has laid out clear examples and definitions of macroscales in different system types [8,12,22].

Note that our handling of causal emergence here is simpler than definitions that either search across the set of macroscales [8], or estimate the results of such a search [55]. We can leave such issues aside in our model system by simply grouping each side of the bipartition and using that as the macroscale. Specifically, we use a microscale with N=16 microstates $\Omega_{micro}=\Omega_{A}\cup \Omega_{B}=\left\{0000,0001,0010,0011,0100,0101,0110,0111\right\}\cup \left\{1111,1110,1101,1100,1011,1010,1001,1000\right\}$ and two macrostates $\Omega_{macro}=\left\{\mathrm{ON},\mathrm{OFF}\right\}$ defined by the coarse-graining function $h:\Omega_{micro}\to \Omega_{macro}$, with $h(\Omega_{A})=\mathrm{ON}$ and $h(\Omega_{B})=\mathrm{OFF}$ (Figure 2B).

This coarse-grains the bipartite model into a simple two-state system at the macroscale, which trades off between the two macrostates (essentially, the same dynamics as a NOT gate with a self-loop). This macroscale is deterministic (each macrostate transitions solely to the other) and non-degenerate (each macrostate has only one possible cause). This means that, for the bipartite model, the macroscale is deterministic, non-degenerate, and dynamically consistent no matter the underlying microscale. Conceptually, its dynamical consistency comes from how, no matter the underlying microstate, the bipartite model always transitions to a different microstate on the other “side” of the bipartite model in the next timestep, and the two macrostates simply are the two sides. This allows us to compare a consistent macroscale against parameterizations of noise, like increases in indeterminism and degeneracy at the microscale, while keeping the macroscale fixed. Additionally, the stationary intervention distribution, maximum-entropy distribution, and local intervention distribution can be easily assessed at the macroscale in the bipartite model, ensuring clear comparisons.

The bipartite model has a further advantage. In previous research on causal emergence, there is a further check of candidate macroscales to ensure they are dynamically consistent with their underlying microscale. This means that the macroscale is not just derivable from the microscale (supervenience) but also that the macroscale behaves identically or similarly (in terms of its trajectory, dynamics, or state transitions over time). Mathematical definitions of consistency between scales have been previously proposed [12], and later work has also proposed similar notions to consistency by using the “lumpability” of Markov chains to analyze the issue of dynamical consistency between microscales and their macroscales [31]. Here, however, we can again eschew this issue. This is because the macroscale for the bipartite model we use automatically ensures dynamical consistency.

For these reasons, we focus on a simplified definition of causal emergence in the form of instances of macroscale causation in our bipartite model without a search across scales or an accompanying causal apportioning schema that distributes out macroscale causation across multiple scales, as in [56]. Here causal emergence (CE) is computed as merely the difference between the macroscale causal relationships and the microscale causal relationships, with respect to a given measure of causation.

$$CE=CS_{macro}-CS_{micro}$$

If CE is positive, there is causal emergence. This can be interpreted as the macroscale doing more causal work, being more powerful, strong, or more informative, depending on how the chosen measure of causation is itself interpreted. A negative value of CE indicates *causal reduction*.

## 3. Results

### 3.1. All Measures of Causation Are Sensitive to Noise

**Figure 3:**
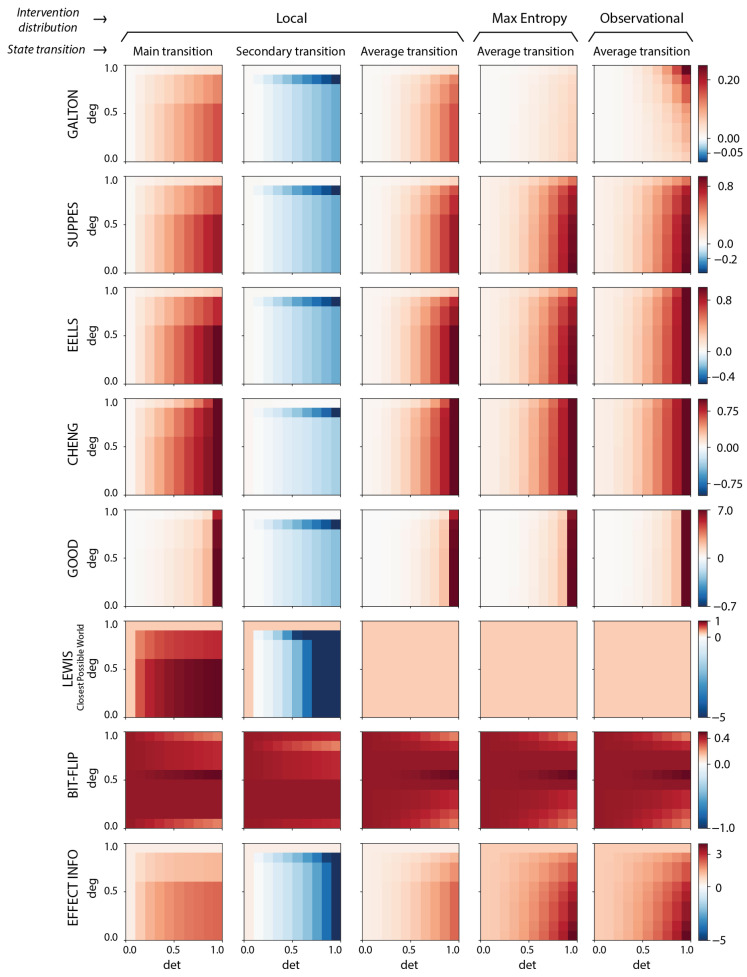
**Behavior of the causation measures in the model system.** Heatmaps of causal strength (or whatever similar interpretation the measures have) are shown for all measures (rows) calculated for the microscale of the bipartite Markov chain model with n=16 microstates and 8 states in each macro-group ($\Omega_{A}=\left\{0000,0001,0010,0011,0100,0101,0110,0111\right\}$ and $\Omega_{B}=\left\{1111,1110,1101,1100,1011,1010,1001,1000\right\}$), at different values of the determinism and degeneracy parameters (see Appendix A.1 for a detailed description). Positive values indicate presence of causal strength and are depicted in red, while negative values correspond to what is known as preemptive or negative causation and are shown in blue. Each measure was calculated for different state transitions in the bipartite model: a main transition, where a strong causal link is thought to be present (0000→1111); a secondary transition, where the causal relationship is supposedly weak (0000→1110); and the average across all state transitions. The measures were also calculated using different intervention distributions (local, maximum-entropy, and observational). For each measure (row), a common scale is used (shown in the color bar). The heatmaps computed for the causal primitives can be found in Figure A2.

To demonstrate the consilience between measures of causation, as well as their underlying causal primitives, we study their behavior in the model described in Section 2.5 under different parameterizations of noise in the form of indeterminism and degeneracy. Due to how we paramaterize determinism and degeneracy, we can simplify looking at every single transition in the model into just two. This is because any given state has a *main transition*, which is the transition of highest probability (e.g., 000→111 in Figure 2A), and its exhaustive set of *secondary transitions*, which are the lower probabilities of transitions (e.g., 001→111 in Figure 2A). When the probability of main transitions equals that of the secondary transitions, the system is maximally indeterminate, since all state transitions are a random choice (maximum noise of prediction). This is what is occurring along the det (determinism axis) in Figure 3. When main effects are stacked on top of a given target, this is increasing the deg (degeneracy axis) (maximum noise in retrodiction). The precise nature of this parameterization and how it reflects the determinism and degeneracy is discussed in Appendix A.1.

We apply the measures of causation in Section 2 in both a state-dependent and a state-independent manner, since both are common throughout the literature on causation [3,57,58,59,60,61]. That is, we examine the behavior of the measures on specific individual transitions (such as identifying strong or weak causes) but also their expectation averaged across all transitions, thus covering both individual and global causal approaches.

Our expectation was that measures of causation should peak in their values when determinism is maximized and degeneracy is minimized. And indeed, that is what we find in the bipartite model across all the measures of Section 2.3, but individually and also in global expectation (with the sole exception of the bit-flip measure, but this may be a function of our arbitrary state-labeling, since it is sensitive to that).

Furthermore, we consider different intervention distributions used to probe counterfactual space: the maximum-entropy distribution, where all states are equally and exhaustively probed; the stationary distribution, where the states are weighted according to their frequency of occurrence in the long-term dynamic of the system; and the local perturbation distribution, where a subset of the full state space is probed by considering states that are close to the candidate cause according to some criteria of distance (e.g., Hamming distance).

**Figure A2:**
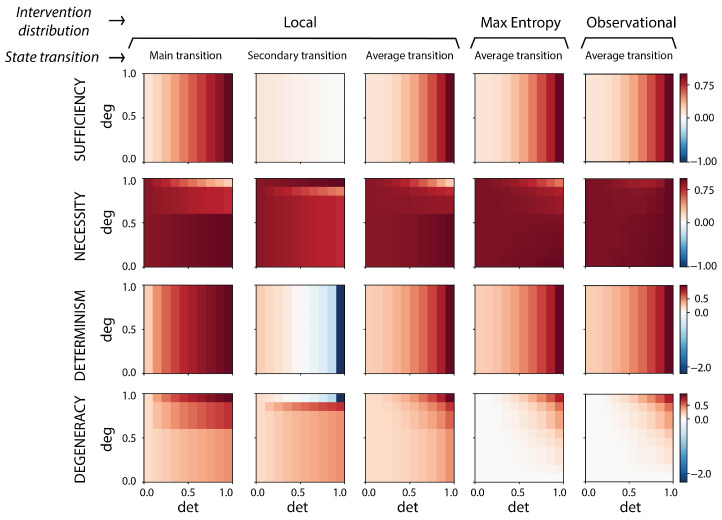
**Behavior of the causal primitives in the model system.** Shown along the rows are the heatmaps of the causal primitives, i.e., sufficiency, necessity, determinism, and degeneracy, for different values of the model’s degeneracy and determinism parameters. In the first three columns, the maximum-entropy distribution is used to calculate the causal primitives and average the across transitions. In the first two columns, the causal primitives are assessed for single state transition between a cause and an effect: first, between a cause and its main effect (0000→1111), generally a strong causal link; and second, between a cause and a non-principal effect, which generally we would expect to have a weaker causal strength (0001→1111). In the third column, we show the simple average of the causal primitives across all the state transitions. In the fourth column, the average of the causal primitives is shown, but using the stationary distribution to estimate the primitives and also compute the average. In the last column, the causal primitives are averaged across all transitions, computed using local perturbations.

The vast majority of the measures of causation increase with the determinism of the model and decrease as the model becomes more degenerate (Figure 3). Moreover, the system-level behavior of the causation measures, i.e., the average across all state transitions, is dominated by that of the main transitions, which is consistent with the pre-theoretic notion that these transitions constitute the main causes. Note that the results shown are using local perturbations, but using the other intervention distributions leads to qualitatively similar results, i.e., across both the maximum-entropy distribution and the observational distribution (shown in Figure A2). This indicates that local perturbations may provide an efficient surrogate for computing causal powers, at least in cases where causal powers are “locally distributed” across nearby states (according to some distance metric), without either (a) relying on the exhaustive exploration of counterfactual space that may contain intuitively nonsensical counterfactuals, or (b) using an observational distribution that reflects the system’s dynamics rather than its causal structure.

### 3.2. All Measures of Causation Assessed Show Causal Emergence

**Figure 4:**
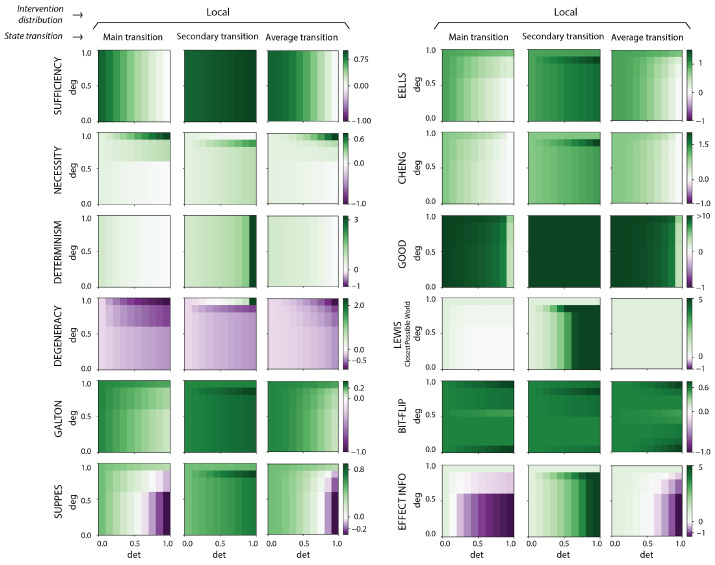
**Causal emergence is widespread across choice of measure of causation and intervention distribution.** Heatmaps of causal emergence (CE) and causal reduction (CR) are shown for all measures of causation and causal primitives computed in the bipartite Markov chain model. Causal emergence is calculated as the difference between the causation metric calculated at the macroscale and at the microscale, such that positive values (green) amount to CE and negative values (purple) to CR. CE/CR is assessed using a local intervention distribution, in which a subset of counterfactuals is created by perturbing the cause around “close” states. In each of the three columns, CE/CR is assessed over different state transitions of the system: a main transition with a strong causal strength (0000→1111), a secondary transition with a weak causal strength (0000→1110), and the expectation over all state transitions. The joint probability P(c,e) used to compute the expectation is obtained using the transition probabilities P(e∣c) and the stationary intervention distribution $P_{obs}\left(c\right)$. For each measure (row), a common scale is used (shown in the color bar). The causal emergence across the full combinations of intervention distributions and transitions can be found in Figure A3.

**Figure 5:**
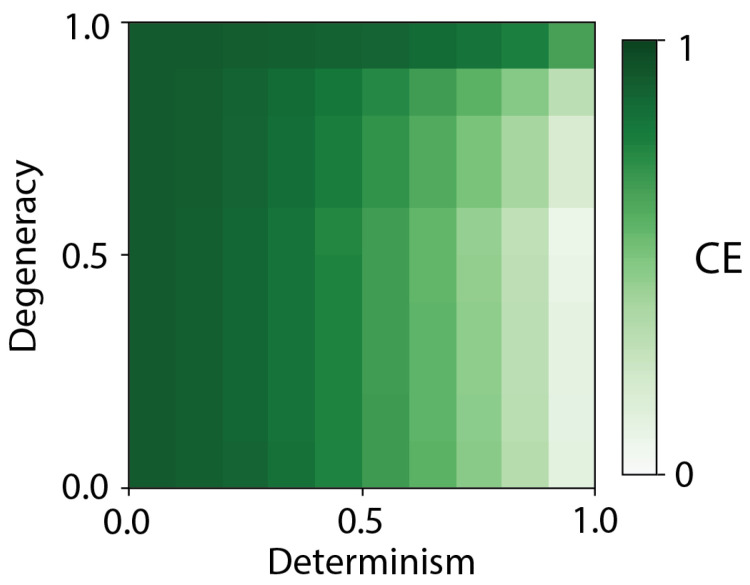
**Causal emergence occurs when the microscale is noisy.** Average behavior of causal emergence across the eight causation measures and four causal primitives for the bipartite Markov chain model. All twelve metrics were normalized to range from −1 to 1 by dividing each metric by its maximum absolute value of CE/CR, and then combined through a simple average at each value of determinism and degeneracy (based on changes to model connectivity discussed in Figure 2B). While measures may have different relative CE values at any one point, across measures values tracked the amount of noise (indeterminism) and common causes (degeneracy), with more noisy and degenerate regimes being associated with higher degrees of causal emergence, ranging from low or near-zero CE (light green) to high CE values (dark green), as shown in the color bar.

Taking into consideration different transitions in the model and employing different intervention distributions, all measures of causation exhibited instances of causal emergence, as shown in Figure 4). Exactly as would be predicted by the idea that macroscales provide error correction of noise in causal relationships, causal emergence is greater when determinism is low and degeneracy is high in the microscale across the set of measures (see Figure 5). Moreover, causal emergence occurred most prominently in secondary transitions, where causal strength (broadly referring to the interpretation of the causal measures) of the microscale was shown to be lower due to noise, compared to main transitions. There were even cases of what might be termed “infinite causal emergence”, wherein a microscale transition acted as a preventative cause (due to a negative value for the causal measure) while the macroscale transition had a positive value, according to the same measure. Additionally, at the global system level, such as in the expectation, there was also a significant amount of causal emergence in various system architecture domains (particularly those with more uncertainty).

**Figure A3:**
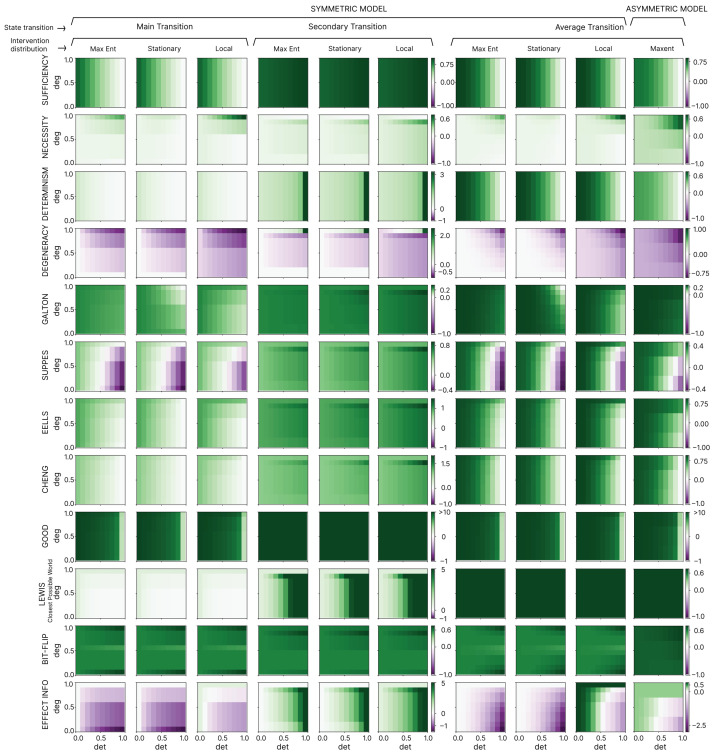
**Causal emergence is generally invariant to intervention distribution choice, as well as symmetry breaking.** An expanded version of Figure 4, including three different choices of intervention distributions, as well as what happens when the bipartite model is not perfectly symmetric. Heatmaps of causal emergence (CE) and causal reduction (CR) are shown for all measures of causation and causal primitives computed in the bipartite Markov chain model. Causal emergence is calculated as the difference between the causation metric calculated in the macroscale and in the microscale, such that positive values (green) amount to CE and negative values (purple) to CR. CE/CR is assessed using a maximum-entropy distribution, a local intervention distribution, and the observational distribution. CE/CR is also assessed over different state transitions of the system: a main transition with a strong causal strength (0000→1111), a secondary transition with a weak causal strength (0000→1110), and the expectation over all state transitions. The joint probability P(c,e) is used to compute the expectation using the observational intervention distribution $P_{obs}\left(C\right)$. For each measure (row), a common scale is used (shown in the color bar). In the last column, the model was calculated using an asymmetric version of the bipartite model with $n_{A}=13$ and $n_{B}=3$ states on each macro-group, instead of $n_{A}=n_{B}=8$ used in the rest of the paper.

The ubiquity of causal emergence did not depend on the particular way of performing the intervention distribution (which all measures implicitly require be specified in their application). Cases of causal emergence were present across all measures of causation calculated using the maximum-entropy distribution, the observational intervention distribution, and the local intervention distribution (see Figure A3), although distributed slightly differently depending on choice, appearing in all but one measure in the symmetric model and all measures in the asymmetric model.

The one exception where causal emergence was absent (at least on average, but not in terms of individual transition contributions) was the effective information calculated using the observational distribution, which is mathematically equivalent to mutual information [62]. This was previously leveled as a criticism of the theory of causal emergence [24,63], proposing that the phenomenon depended strictly on EI’s use of a maximum-entropy distribution, since comparatively the mutual information cannot be higher at a macroscale. However, mutual information is generally not regarded as a causal measure [64], and fails to capture intuitive common-sense cases that measures of causation should satisfy [65] (e.g., like a cycle of causally linked COPY gates, about which the mutual information does not capture causation well, since the mutual information is sensitive to the homogeneity of the initial states—this rules out most proposed measures based on information flows as well, which we do not include for the same reason). Indeed, it is arguable that *no* measure calculated solely from an observed distribution can be a good measure of causation [53]. Therefore, the widespread appearance of causal emergence across measures of causation, even when using the observed distribution, indicates both its ubiquity and independence from restrictive mathematical assumptions like a maximum-entropy requirement (at least, across most measures).

To confirm that our findings were not driven by the symmetry of the bipartite model (i.e., the equal size of the macro-groupings, $n_{A}=n_{B}$), we evaluated causal emergence in an asymmetric version of the model. This analysis yielded similar results (Figure A3).

**Figure A4:**
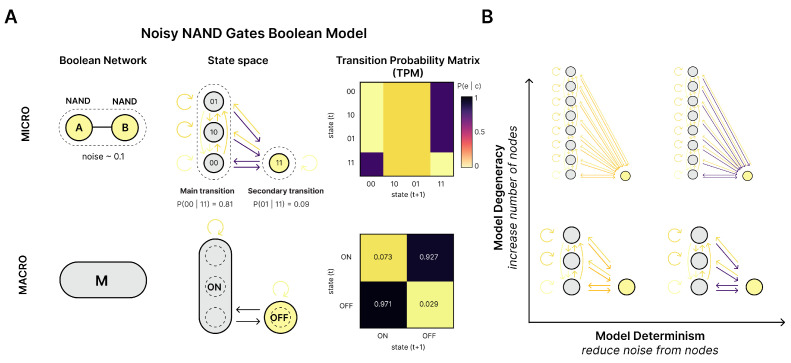
**Boolean model of interacting NAND gates.** (**A**) *Noisy Boolean network model of interacting NAND gates.* In this depicted example, two micro-nodes A and B form a recurrent network composed of two noisy NAND logic gates. When both units are one (11), the system transitions with high probability (0.9) to the all-zero state (00), and with lower probability to other states. Conversely, when not all units are one (gray states), the system transitions with high probability to 11, and with low probability to the rest. A main transition from 11 to 00 is highlighted, along with a secondary transition from 11 to 01. At the macro level, a single node *M* is defined with ON={11} and OFF={00,01,10}, yielding a coarse-grained dynamic between macrostates. (**B**) State-space visualizations of the NAND model across increasing *degeneracy* (via increased number of nodes) and *determinism* (via reduced noise).

**Figure A5:**
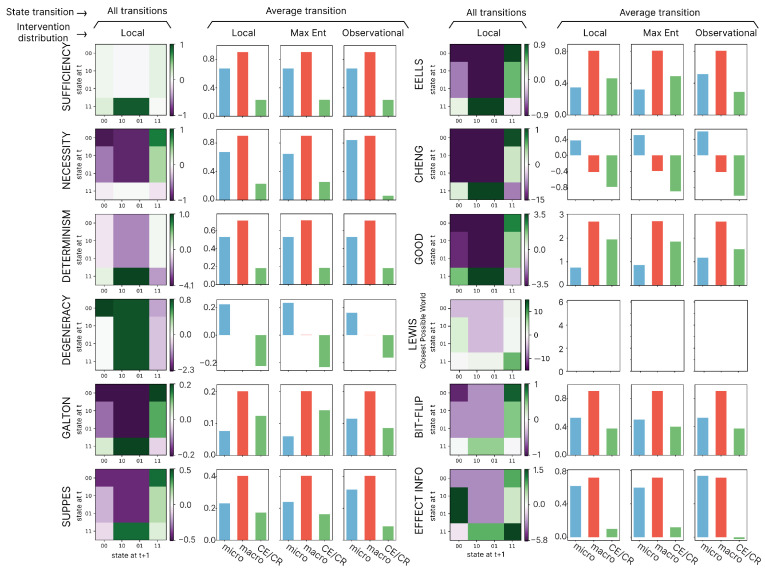
**Causal measures and causal emergence in a Boolean network of two interacting noisy NAND gates.** Causation measures computed for the two noisy NAND logic gates system (with noise set to 0.1). The heatmaps show the degree of causal emergence (in green), or causal reduction (in purple), i.e., the difference between the causation measure calculated at the macroscale and the microscale, for all state transitions of the model system. The bar graphs show the causation measures at the microscale (blue), macroscale (red), and the amount of causal emergence/reduction (green), averaged across all state transitions. These values were computed using different intervention distributions to assess the counterfactuals: the maximum-entropy distribution (all states are uniformly sampled); the stationary distribution (states are sampled according to the observed distribution of the dynamics of the model); and the local distribution (the candidate cause is locally perturbed, so that “close” counterfactuals are sampled). The average across transitions was computed as the joint expectation of the measure CS(c,e) using P(c,e), which is obtained from the transition probability matrix (TPM) and the respective intervention distribution, except for the local intervention, where the observational distribution was used (using maxent led to similar results).

To complement our analysis and ensure that our results are not confined to the bipartite system or limited to models with abstract state transitions with no physical realization, we extended the analysis to a Boolean network model of causally interacting gates, composed of noisy, interconnected NAND gates (Figure A4). In the case of two nodes (Figure A4A), when both units are ON (11), the system transitions with high probability (e.g., 0.9, noise = 0.1) to the all-OFF state (00), and with lower probability to other states. Conversely, when not all units are ON (i.e., 00, 01, or 10), the system transitions with high probability to 11, yielding a bistable microdynamic shaped by logical interactions and noise. A macroscale variable *M* is defined by grouping the four microstates into ON=11 and OFF=00,01,10, inducing a coarse-grained, binary macro-dynamic. Figure A5 shows the resulting values of each causal measure, computed at both the micro and macro levels, as well as the degree of causal emergence or reduction at each state transition. Most measures display positive causal emergence, either at the average level or at the level of single transitions, thus reinforcing the generality of the phenomenon across structurally different causal systems.

**Figure A6:**
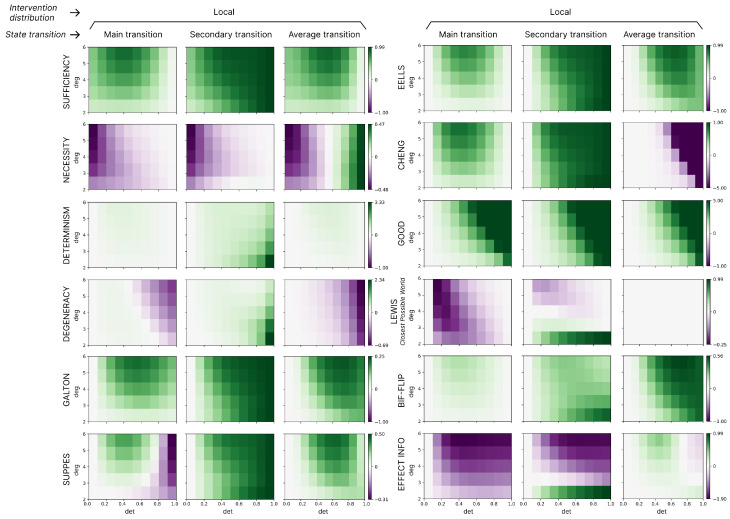
**Causal emergence in the Boolean network of interacting noisy NAND gates.** Heatmaps show causal emergence (CE) and causal reduction (CR) for all causal primitives and causation measures applied to the Boolean network model composed of interacting noisy NAND gates. As in the main analysis (Figure 4), CE is computed as the difference between the macroscale and microscale values of each measure, with positive values (green) indicating causal emergence and negative values (purple) indicating causal reduction. All evaluations use a local intervention distribution, where counterfactuals are restricted to perturbations of nearby states. Each column corresponds to a different transition type: a main transition with strong causal influence (e.g., 11→00), a secondary transition with weaker influence (e.g., 11→01), and the average over all transitions weighted by the stationary distribution. Color scales are individually normalized across rows. This analysis complements the one presented in Figure 4, indicating that, even when systems differ in construction, and degeneracy is increased in a different way (here, adding gates to the network), causal emergence is detectable in many cases and conditions across measures.

Importantly, this model also allowed us to manipulate determinism by adjusting the noise level in the logic gates, and degeneracy by increasing the number of nodes (and thus the possible many-to-one input–output mappings), as illustrated in Figure A4B. In Figure A6, we systematically vary the determinism and degeneracy of the Boolean network by adjusting the noise level and the number of micro-nodes, respectively. Unlike the bipartite model, where the macro TPM was held fixed across parameter sweeps, here the macroscale dynamics themselves change with the underlying microscale configuration. This means we cannot isolate the effect of changing microscale determinism and degeneracy on causal emergence as cleanly (i.e., more degenerate models have more micro-nodes, in this case). Nevertheless, the results echo a key qualitative trend observed earlier: causal emergence tends to appear when the microscale exhibits high degeneracy and low determinism. This convergence across models—despite structural differences, variable macro-dynamics, and even how degeneracy itself is being varied—supports the robustness of causal emergence as a general property.

## 4. Discussion

Disparate measures of probabilistic causation at first appear different, and are said to measure things like causal strength, influence, power, informativeness, predictiveness, or work (depending on the details of the chosen measure of causation and the terminology). However, we show that causation is not itself a primitive notion but can be decomposed along two dimensions (a finding in agreement with speculations by previous authors [1,66]). In the philosophical literature these two dimensions are referred to as sufficiency and necessity. As we show, across many probabilistic measures of causation, these two dimensions can be identified in the terms making up the measures. In info-theoretic measures, these dimensions take the functional forms of determinism and degeneracy, respectively. Across almost every measure of causation we examined, the two primitives (sufficiency and necessity), or alternatively their info-theoretic formulations (determinism and degeneracy), are explicitly put in some relationship, often that of a difference, ratio, or trade-off (Figure 1B), with most measures systematically increasing with sufficiency and necessity, or with determinism and non-degeneracy (Figure 1C). Successful measures of causation, many of which have been independently proposed and later discovered by other authors, are always sensitive to both dimensions (which we dub “causal primitives”). In other words, there is significant consilience when it comes to mathematical measures of causation: rather than being a simple notion, causation corresponds to the joint presence of the two dimensions causal sufficiency and necessity, or relatedly, of causal determinism and lack of causal degeneracy. While it remains unknown how much measures may ever disagree in some possible system or circumstance (and how to interpret that), we have shown that their sharing basic terms entails that measures of causation are jointly sensitive to noise in the form of uncertainty over the future (indeterminism in the effects) and uncertainty over the past (degeneracy in the causes).

We are not the first to point out that causation has two dimensions: for instance, Judea Pearl [1] states: “Clearly, some balance must be struck between the necessary and the sufficient components of causal explanation.” Also J. L. Mackie, although not proposing a quantitative measure of causal strength, famously considers both a necessity and a sufficiency aspect in his proposal of an INUS condition that causes should satisfy; namely, being an (i)nsufficient but (n)ecessary part of a condition which is itself (u)nnecessary but (s)ufficient for an effect to occur [66]. However, to our knowledge, this is the first time that (i) the connection between probabilistic and info-theoretic causal primitives has been established and (ii) a full set of popular measures has been assessed in this light, and so we state explicitly: substantial consilience in measures of causation indicates we should expect measures of causal strength to be based on *both* causal primitives.

Our results support this inference. The only measure that lacked an explicit basis in causal primitives was the bit-flip measure—it therefore arguably does not behave appropriately as a measure of causation, and indeed, it is the least similar in its behavior with the other measures, indicating the importance of the shared reliance on causal primitives for well-tuned measures of causation and appropriateness for detecting causal emergence and causal reduction (as the bit-flip measure universally detects positive CE across the conditions we examine; see Figure 4). Additionally, it is worth noting that the Lewis measure, while scaling similarly to other measures for individual causes, averaged out to zero in both of our models (see Figure 3 and Figure A5), indicating that it too may be an inappropriate measure of causation.

These findings set a firm foundation for understanding causal emergence: macroscales perform error correction over their underlying microscale causal relationships, improving the measures of causal strength (or influence, power, etc.) either by decreasing indeterminism (increasing sufficiency) or decreasing degeneracy (increasing necessity) (Figure 5). Here we have shown that causal emergence (here, simplified as just instances of macroscale causation) is identifiable within popular measures of causation with independent origins in diverse fields. Across the more than a dozen measures of causation we examined, all were highly related by sharing similar basic terms (which we dub “causal primitives”); many turned out to be identical rediscoveries; and all demonstrated cases of causal emergence in a bipartite model system in conditions of high uncertainty over state transitions (low determinism, high degeneracy). This was true across a large number of possible assumptions of how exactly those measures were applied. This provides context for previous research, which has already shown causal emergence using more complex information-theoretic measures of causation, like effective information [8], the integrated information [20], and also, recently, synergistic information [33]. Interestingly, we find that effective information, despite being the original measure proposed to capture causal emergence, is the most conservative measure in our sample.

It is worth noting that the measures of causation we examined need a space of counterfactuals in order to apply the measure. Here, we represent this choice mathematically using an intervention distribution. In the systems we study, we find that causal emergence is relatively invariant across choice of intervention distribution, indicating that it is a robust phenomenon. While the choice of intervention distribution in the majority of measures does not affect the possibility of causal emergence, we advocate for our notion of “local interventions” [10] as being a step forward for mathematical measures of causation, as it offers a compromise between a maximum-entropy approach (all possibilities considered) and a minimal-difference approach (only the closest possibility is considered).

Despite the ubiquity of causal emergence across measures and background conditions, the existence of emergence itself is not trivially guaranteed. Rather, it is a function of system architecture or dynamics. As we have shown, in deterministic system mechanics, causal reduction dominates. However, in scientific models these conditions are quite rare, as science deals with mainly open systems exposed to outside uncertainty or, alternatively, systems with inherent uncertainty; in both cases limited by the resolution of the measurement apparatus. Even systems with irreducibly small amounts of noise can have that noise amplified into significant uncertainty after dynamical iteration [67]. Therefore, we expect instances of causal emergence to be common across the many scales and models of science

A limitation of this paper is that it only addresses the aspect of causal emergence related to identifying instances of macroscale causation (when the “macro beats the micro”, according to some specified causal measure). As a result, we do not address the question of how to identify in general the natural scale at which causation takes place (wherein “natural” means optimal according to some criteria). For example, a computer might (or might not) have a natural scale at the level of its operating code. Earlier research has used the effective information and integrated information to search for natural scales by finding the spatiotemporal scale where the value of the chosen causal measure is maximized [8,20]. More recently, a novel framework for causal emergence was put forward: Causal Emergence 2.0 [56]. This posits a causal apportioning schema that distributes out the gains in the causal primitives described herein across scales.

Another important limitation of our approach is that it presupposes full knowledge of the system’s causal dynamics, as encoded in a transition probability matrix. This stands in contrast to causal inference frameworks, such as those developed by Pearl, which aim to infer or represent causal relations from partial observational and interventional data. Further work is necessary to extend our analysis to settings where the causal model is only partially known or must be inferred, potentially bridging our framework with causal discovery and structural causal model approaches.

The development of complex systems science was based on novel insights into how complexity can arise via iteration of simple rules [68,69,70]. But rather than being based on one fully agreed upon measure of complexity, the subfield has always been based around a family of measures of complexity [7]. The development of a science of emergence should similarly be based on causal primitives (captured and set in relation by the family of measures of causation) along with the noise-minimizing properties of macroscales. Ultimately, this work provides a necessary toolkit for the scientific identification of macroscale causation, which has wide-ranging implications for optimal modeling choices, interventions, and scientific explanations.

## Figures and Tables

**Figure A1:**
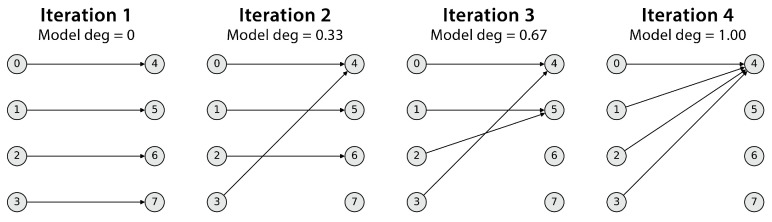
**Visualization of steps in the algorithm to increase degeneracy in the bipartite model.** Iterations of the algorithm for the bipartite Markov chain state space with n=8 states. States are labeled from 0 to n−1, with states in the left column belonging to group *A* and on the right to group *B*. The function $f_{A}:\Omega_{A}\to \Omega_{B}$ maps states in group *A* to states in group *B*, associating every candidate cause $c\in \Omega_{A}$ to a main effect $f_{A}\left(c\right)=e^{*}$. At each iteration, a main effect is lost (i.e., the image $f\left(A\right)\in \Omega_{B}$ loses an element) as an arrow is moved an effect with the least number of arrows (with at least one arrow). The main effects of each cause progressively overlap until they are over a single state in group *B*. For $f_{B}$ the same algorithm is applied, but with the states *A* and *B* reversed.

## Data Availability

The code used for calculating the measures of causation as well as assessing causal emergence on the bipartite Markov chain model is available at https://github.com/renzocom/causal_emergence (accessed on 7 July 2025).

## Appendix A

### Appendix A.1. Parameterizing Determinism and Degeneracy in the Bipartite Model

What follows is a detailed description of how we algorithmically vary det and deg in the bipartite Markov chain model. First, we label the $2^{N}=n$ states with binary strings and divide them into two groups *A* and *B* of size $n_{A}$ and $n_{B}$, respectively. For now, let us consider the symmetric case where $n_{A}=n_{B}$. For example, with N=3 we have states $\Omega =\Omega_{A}\cup \Omega_{B}=\left\{000,001,010,011\right\}\cup \left\{111,110,101,100\right\}$) (Figure 2A, top). The model’s dynamics is governed by a transition probability matrix, where for a given state c∈Ω the system is in, it can transition to a state e∈Ω, with a probability given by P(e∣c), such that any state transition defines a cause and effect pair (Figure 2A, bottom). Each cause state is paired to a main-effect state in the opposite grouping through a mapping $f_{A}:\Omega_{A}\to \Omega_{B}$. A complementary mapping is given $f_{B}\left(s\right)=f_{A}\left(s↑\right)↑$, where ↑ is the state obtained by inverting all bits (e.g., ↑100=011) (this is equivalent to reflecting the arrows in Figure 2A along the vertical axis). For a given state $c\in \Omega_{A}$ we have

(A1) $$P\left(e|c\right)=\left\{\begin{array}{cc} p, & \mathrm{if}\;e=f_{A}\left(c\right)\;\mathrm{and}\;e\in \Omega_{B}\\ \frac{\left(1-p\right)}{N_{B}} & \mathrm{if}\;e\neq f_{A}\left(c\right)\;\mathrm{and}\;e\in \Omega_{B}\\ 0 & \mathrm{otherwise} \end{array}\right.$$

If $c\in \Omega_{B}$, we simply interchange *B* for *A* in the definition above. 0≤p≤1 is the parameter which controls the determinism of the system, by concentrating or diluting the probability over the main effect (vs. the secondary effects) of a given cause. Essentially, we are simply narrowing or widening the “scope” of possible effects from a given state in order to increase or decrease the determinism, respectively.

In order to parametrically vary the degeneracy of the model, we change the mapping $f_{A}$ (and its complement $f_{B}$), going from zero degeneracy, where $f_{A}$ is injective, (maps different cause to different main effects according to $f_{A}\left(s\right)=↑s$), to maximum degeneracy, where all causes in one group map to a single effect in the other group (Figure 2B). To increase the degeneracy in a stepwise manner we use the following algorithm: we chose the “poorest” effect, i.e., the one with the least number of main causes ($argmin|f^{-1}\left(e\right)|$) but with at least one main cause, and move all its main causes to the next poorest effect. In this way, we progressively re-wire the system until all causes map to only one effect and maximal degeneracy is achieved. Essentially, we are simply moving main effects on top of one another sequentially—this increases the degeneracy (and decreases the necessity). A visual example of this can be seen in Figure A1.

However, it should be noted that our algorithmic methods for varying the determinism and degeneracy do not automatically ensure that it is changing the causal primitives as expected. This is because the algorithmic way of varying determinism and degeneracy (by varying the probabilities between main effects and second effects, and stacking main effects on top of targets, respectively) does not match one-to-one with the underlying mathematical properties of determinism and degeneracy. This is because there is no way to smoothly vary the actual mathematical properties in a simple algorithmic manner.

However, when the causal primitives are computed over different parameters of the model and we consider their global behavior averaged across all transitions in the state space, the algorithmic determinism and sufficiency indeed scale with their mathematical counterparts. Similarly, the degeneracy primitive scales proportionally to the model’s degeneracy parameter, while necessity scales inversely (Figure A2). Note that while determinism and sufficiency are independent of the model’s degeneracy parameter, degeneracy and necessity are sensitive to the model’s determinism parameter, simply due to its algorithmic construction. These results validate the bipartite model’s capacity to explore the behavior of the causal measures for different combinations of causal primitives, as modulated by the model’s degeneracy and determinism parameter; although, due to the inability to vary degeneracy without varying the determinism, it does not perform this over a perfectly symmetric manifold.

It is also interesting to note how the causal primitives behave differently for specific transitions with strong and weak causal strengths. The main transitions exhibit high sufficiency, determinism, and necessity and low degeneracy (Figure A2, first column); in particular, in regions of high determinism and low degeneracy of the parameter space of the model. This behavior dominates and appears at the level of the average quantities across all transitions (Figure A2, last three columns). The secondary transitions show lower values in general for all causal primitives, coherent with the notion that they are endowed with weaker causal powers. In line with this, the determinism and degeneracy of the secondary transitions vary in the opposite manner to a main transition, peaking when the determinism of the model is low, and the degeneracy is high (Figure A2, second column).
